# Improving Equitable Access to Disability Services and Support for Children With Neurodevelopmental Disabilities: Service Provider and Decision‐Maker Perspectives

**DOI:** 10.1111/cch.70213

**Published:** 2025-12-29

**Authors:** Patricia Basualto, Angela M. Senevirathna, Ashish Seth, Gina Dimitropoulos, Jennifer D. Zwicker

**Affiliations:** ^1^ Faculty of Kinesiology University of Calgary Calgary Alberta Canada; ^2^ School of Public Policy University of Calgary Calgary Alberta Canada; ^3^ Departamento de Kinesiología, Escuela de Ciencias de la Salud, Facultad de Medicina Pontificia Universidad Católica de Chile Santiago Chile; ^4^ Department of Social Work, Faculty of Social Work University of Calgary Calgary Alberta Canada; ^5^ Department of Psychiatry Mathison Centre for Mental Health Research & Education Calgary Alberta Canada

**Keywords:** access, disability services, disparity, neurodevelopmental disability, qualitative

## Abstract

**Background:**

Disparity in access to services for children with neurodevelopmental disabilities is a modifiable barrier to improving health, functional outcomes and social participation. Unfortunately, children and youth with neurodevelopmental disabilities face many barriers in accessing education and disability services and support. This study aimed to describe the perspectives of service providers and decision‐makers about disparities in access to disability services and support for children and youth with neurodevelopmental disabilities and their families.

**Methods:**

Utilizing a qualitative descriptive methodology informed by a pragmatic epistemological approach, we interviewed 16 individual service providers and decision‐makers from childhood education and disability services across ministries in British Columbia. Purposeful and maximum variation sampling was used to guide the selection of participants. An advisory council provided patient and family‐oriented perspectives and reviewed, piloted and refined the semi‐structured interview guide. Data was analysed with inductive thematic analysis.

**Results:**

System barriers and facilitators to accessing education and disability services and support were identified both through the navigation journey and across ministries. To address these barriers and leverage facilitators, policy and program design recommendations were identified, including an approach for framing policy as a ‘wider door’ and developing a holistic system that ‘works for everybody’.

**Conclusions:**

Systemic barriers and facilitators intertwine within the system before, during and after engagement with families and children. This creates a complex delivery environment, hindering the equitable provision of disability services and support. The findings provide a systemic and intersectoral overview of the interconnected challenges encountered while navigating the system, relevant to many jurisdictions.

## Summary

- Education, health and social disability policy and program design must bridge the know‐do gap in implementation from the outset. This study describes the perspectives of individual service providers and decision‐makers on what works and under what conditions within a system of disability services and supports.
- Participants described how education policy and implementation of disability services and support create a ‘narrow’ door for eligibility and access to a fragmented system, marked by multiple barriers and facilitators throughout the disability system of services and support.
- This study provides a systemic and intersectoral overview of the interconnected challenges children with NDDs and their families face before, during and after accessing services and support in the educational, health and social sectors.
- It is also the first to report contextual differences in how environmental factors challenge the navigation process within a disability system of services and support. It presents an integrated participants' perspective for policy and program change recommendations aimed at the overall structure of the disability services and support system.

**Abbreviations**
ACCESS: Assessing the Continuum of Care and Eligibility for Services and Supports
AF: Autism Funding program
AHP: At Home Program
ASD: autism spectrum disorder
ASL: American Sign Language
bc
: British Columbia
BCAAN: British Columbia Autism Assessment Network
CYSN: Children and Youth with Support Needs
MCFD: Ministry of Child and Family Development
MEdC: Ministry of Education and Childcare
MoH: Ministry of Health
NDDs: neurodevelopmental disabilities
NSS: Nursing Support Services
OT: occupational therapist
PT: physical therapist
SLP: Speech and Language Pathologist

## 1 Background

Most children with neurodevelopmental disabilities (NDDs) experience functional and/or behavioural challenges that contribute to poorer physical and mental health outcomes compared to their peers without NDDs (Prasad and Corbett 2021; Miller et al. 2013; Naik et al. 2023; Namazzi et al. 2019). They are also at a greater risk of lower educational attainment and reduced economic opportunities later in life (Berrigan et al. 2023; Zwicker et al. 2017). These disparities are closely linked to the impact of NDDs—a heterogeneous group of conditions impacting brain development—on the acquisition and integration of early developmental skills, which can impair daily life activities and restrict participation across the life course (Stein et al. 2020). In addition, caregivers of children and youth with NDDs often experience a substantial burden of care, compounded by difficulties in accessing and navigating complex service systems (Arim et al. 2019; Craig et al. 2016).

Children and youth with NDDs are less likely to achieve their full potential when disability services and systems do not address their needs. Ninety percent of individuals with a NDD need access to services and professional support spanning education, health and social services (Berrigan et al. 2023). Still, the lack of access to these services is a critical gap impacting child development and the quality of life (Dutton et al. 2018; Sullivan et al. 2011; Nicholas et al. 2018; Edwards et al. 2022) for children and their families.

Canada has committed to providing services to facilitate full participation in society for individuals with disabilities by ratifying the United Nations Convention on the Rights of Persons with Disabilities (Mason et al. 2022). To fulfil its commitments, a large variety of programs known as ‘disability services and supports’ are delivered at a provincial level in the health, educational, financial and social sectors. Nonetheless, as in many other signatory countries, there is a gap between the federal commitment and provincial implementation of disability support and services (Finlay, Ragot, et al. 2023). Each provincial government has the autonomy to develop the laws, regulations and intersectoral implementation of disability services and supports. This autonomy results in significant provincial and territorial variation in the landscape of equitable service delivery and use in Canada, with barriers related to the application process (including failures or challenges), lack of awareness about the existence of programs (Finlay, Wittevrongel, et al. 2023), wait times, service availability, cost, eligibility and other factors (Edwards et al. 2022). Compared to the national average, children and youth living in British Columbia (bc) are less likely to access disability services and support (Finlay, Wittevrongel, et al. 2023) and face barriers to accessing services designed to support mental health and neurodevelopmental needs (Edwards et al. 2022).

Access to education, health and social services has been widely studied in the general population, and several theoretical models have been used for the past four decades (Dutton et al. 2018; Aday and Andersen 1974; Andersen et al. 1983; Zablotsky et al. 2019; Meade et al. 2015; Lewis 2009). Few empirical studies have described factors related to access to these services and supports for children and youth with NDDs, with the published studies focusing on families' perspectives on the barriers and facilitators they encounter when accessing disability services and support (Edwards et al. 2022; Finlay, Wittevrongel, et al. 2023; Aguerre et al. 2019; Gaona et al. 2020; Pozniak et al. 2024). Fewer studies have focused on service providers' and decision‐makers' perspectives, which can be important for policy design (Cloet et al. 2022; Jerwanska et al. 2023).

Disparity in access to services for children with NDDs is a modifiable barrier to improving health, functional outcomes and social participation from childhood onwards. To achieve better access to services, policy design must bridge the know‐do gap (Graham et al. 2018) with its implementation in mind from the outset (Pollack Porter et al. 2018). By facing the everyday problems of policy and program implementation, service providers and decision‐makers build practical understandings of what works and under what conditions (Head 2010). This study aimed to describe the perspectives of service providers and decision‐makers about disparities in access to education and health disability services and support in bc for children and youth with NDDs and their families. The research question was: What disparities do service providers and decision‐makers describe regarding children and youth with NDDs and their families' eligibility for and access to services and support? Two specific questions helped describe the perceptions about disparities:

1. What barriers and facilitators do children with NDDs and their families face when navigating disability services and support system?
2. What policy and program design recommendations could be implemented to decrease disparities in eligibility and access to disability services and support?

## 2 Methods

### 2.1 Study Design

A qualitative descriptive methodology (Sandelowski 2000, 2010; Colorafi and Evans 2016), informed by a pragmatic epistemological approach (Morgan 2007, 2014), was used to describe the perspectives of individual service providers and decision‐makers (Bradshaw et al. 2017) regarding disparities in access to disability services and support in bc for youth with NDDs and their families, with the goal of providing a rich description of their experiences. The words participants used to describe the events and meanings they considered accurate were retained during the analysis, thereby obtaining a comprehensive summary of the phenomenon of interest from the participants' perspective in their everyday language (Sandelowski 2000). Semi‐structured interviews were conducted from January 2023 to September 2023, and inductive thematic analysis was used to generate themes through coding. The analysis focused on a rich thematic description of the entire dataset to depict patterns across the perspectives of individuals working as service providers and decision‐makers.

### 2.2 Research Context

The bc provincial government administers disability services and support for children through the Children and Youth with Support Needs (CYSN). It delivers programs across three ministries: the Ministry of Child and Family Development (MCFD), the Ministry of Education and Childcare (MEdC) and the Ministry of Health (MoH) (Table 1). Access to most programs and support requires a confirmed diagnosis as eligibility criteria, which vary depending on the ministry and specific program.

**TABLE 1:** Disability system of services and support. Province of British Columbia.

	Services/programs	Description	Services or support provided	Age	Eligibility criteria
MCFD (CYSN)	Foundational programs^a^	Provide direct support and intervention to children and youth who have, or are at risk of, a developmental delay or disability.	Infant development. Aboriginal infant development. Supported child development. Early intervention therapies. School‐aged therapies^b^.	0–3 years 0–6 years 0–12 years 0–5 years 6–19 years	Direct access (self‐referral). Delivered through contracts by community agencies.
	Specialized provincial services^c^	Provide a range of programs and supports to specific populations.	At home program medical benefits. Autism funding. Community brain injury program. Provincial deaf and hard of hearing services. Provincial outreach and professional support.	0–19 years	Program specific diagnosis or assessment eligibility criteria.
	Family support programs^c^	Provide a range of supports for families of children and youth with a specific diagnosis.	Respite. Behaviour support. Parent support. Homemaker/home support workers. Out‐of‐home living. Family support.	0–19 years	Intellectual disability. Autism spectrum disorder. Those eligible for the at home program.
MEdC	Special education services^d^	To provide additional support in an inclusive education system for students with special needs.	Additional staff. Specialized learning materials. Physical accommodations or equipment. Assessments.	6–19 years	Students must be assessed and have an individual education plan (IEP) in place.
MoH	Nursing support services^e^	To provide care for children and youth who require the scope of practice of a registered nurse for some aspects of their care due to the child/youth's medically complex and fragile health needs.	Assessment, planning and monitoring care in: Delegated care: teaching and supporting school staff to assist children and youth with their diabetes care and tube feeds. Direct care: respite care in the home or community for eligible children and youth.	0–19 years	Complete eligibility assessments for the at home program (MCFD).

Abbreviations: MCFD: Ministry of Children and Family Development; MEdC: Ministry of Education and Childcare; MoH: Ministry of Health.

^a^ Diagnosis not required.

^b^ Diagnosis or assessment required.

^c^ Diagnosis or assessment required; families access these services through a CYSN social worker.

^d^ Funds are provided to boards of education to support the needs of students in neighbourhood school classrooms within their district, or placement in a different educational setting when deemed necessary.

^e^ Referrals to NSS are made by a physician or nurse practitioner who is licenced to practice in British Columbia.

As with many systems worldwide, the system for accessing disability support and services in bc is complex and multisectoral. Figure 2 illustrates the steps in accessing support and services, including awareness, identification/assessment, application and service provision.

### 2.3 Participant Recruitment

Potential participants (Miles et al. 2014) were sampled from people working as service providers or decision‐makers in the childhood disability services and support system (health, educational and/or social). Individual service providers included front‐line workers, program coordinators and managers. Decision‐makers worked at the ministry level. Purposeful (Suri 2011; Patton 2002) and maximum variation sampling (Palinkas et al. 2015) was used to identify and facilitate the selection of participants with experience in providing and managing services and support across the province. This was achieved by examining key dimensions of variation to identify and select participants that differ from one another across health, educational and/or social sectors, as well as at different levels within the system.

**FIGURE 1:**
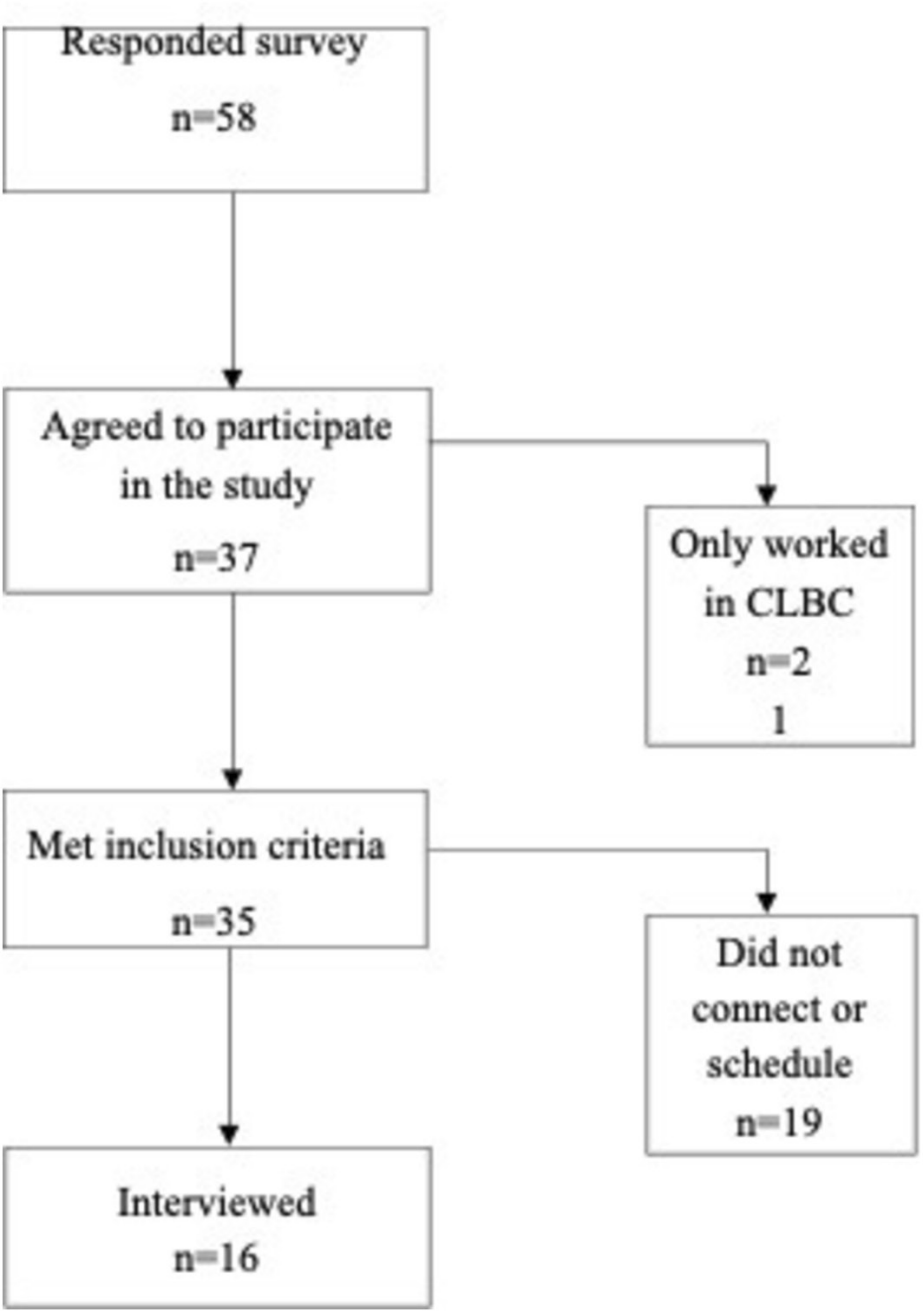
Recruitment. Disability system of services and support. Province of British Columbia.

Prospective interviewees were identified through an internet search on social media and consultation with representatives from our stakeholders (Advisory Council and Kids Brain Health Network). Inclusion criteria covered current or previous experience working with NDD population groups aged 0–18 in bc. The exclusion criteria included not living in bc. A demographics survey administered through Qualtrics was sent to all potential participants to identify their intent to participate and to determine the inclusion/exclusion criteria. An individual invitation was emailed to those who agreed to be interviewed and met the inclusion criteria. The informed consent and a video call connection link were emailed for signature before the interview. From the total number of people contacted (Figure 1), nine service providers and seven decision‐makers from health, social and educational services were interviewed until data analysis showed no new codes and themes. The sampling was guided by seeking information power and trustworthiness from the sample (Colorafi and Evans 2016; Nowell et al. 2017).

### 2.4 Data Collection

A semi‐structured interview guide was developed, including many topics such as service awareness, eligibility criteria, access to services, changing needs, waitlists and family function. These topics emerged from previous interviews (Finlay, Wittevrongel, et al. 2023) in a study aimed at identifying challenges and experiences that children with NDD and their families face when accessing services and support across Canada. Our study Advisory Council piloted the guide and data collection protocol to ensure a patient and family‐oriented perspective, and the interview guide was adjusted based on their feedback.

Interviews were conducted virtually, with durations ranging from 45 to 60 min. The project was presented, along with the opportunity to review questions regarding the consent form and give oral consent to those who did not sign the document in advance. Two interviewers with qualitative research experience were present at each interview: one conducted the interview and interacted with the participant, and a second interviewer took notes on the process. Interviews were audio‐recorded in a digital voice recorder and on the video communication platform. Both interviewers adhered to a pragmatic approach in this study. Audio recordings were transcribed on an ethically approved and authorized platform (Rev.com) and stored on a secure, encrypted University of Calgary drive.

### 2.5 Data Analysis

Inductive thematic data analysis (Bradshaw et al. 2017; Braun and Clarke 2006) was used to derive themes related to experiences providing and administering services for children and youth with NDD and their families, following a six‐phase process. Two researchers independently coded the first six transcripts, compared the codes and reached a consensus regarding names and descriptions of the codes after revisions from senior researchers. The remaining transcripts were coded independently with an agreed‐upon codebook. Themes were identified in the data through an inductive process involving service providers and decision‐makers across sectors. Coding and theme identification were iterated, and new transcripts were added during data collection until enough information power (Malterud et al. 2016) was available in the sample to answer the research questions. The final list of themes and sub‐themes was discussed with senior researchers and presented to the Advisory Council of this project for member checking. Finally, the inductive thematic analysis iterated through within and cross‐interview comparisons, which enabled the report of uniqueness and homogeneity when identifying the shared patterns across the interviews.

### 2.6 Ethics Approval

The Conjoint Faculties Research Ethics Board from University of Calgary (REB 20‐1872) reviewed and approved this study.

## 3 Results

Sixteen people working as service providers and decision‐makers consented to be interviewed. Table 2 summarizes the demographic characteristics of the participants.

**TABLE 2:** Demographic characteristics. Service providers and decision‐makers.

Participant	Program/service	Age range	Years of practice	Communities served
Service provider^a^	CYSN = 7	35–44 years = 11.1%	Min = 2.5 years	Urban = 5
	NSS = 4	45–54 years = 11.1%	Max = 41 years	Rural = 1
	Special education = 3	55–64 years = 44.4%	Mean = 22.1 years	Reserves/settlements = 0
		65–74 years = 44.4%		Urban and rural = 0
				Urban and rural and reserves/settlements = 3
Decision‐maker^a^	CYSN = 7	35–44 years = 28.5%	Min = 1 year	Urban = 1
	NSS = 4	45–54 years = 28.5%	Max = 35 years	Rural = 1
	Special education = 2	55–64 years = 28.5%	Mean = 11.7 years	Reserves/settlements = 0
	Public education = 1	65–74 years = 14.2%		Urban and rural = 1
				Urban and rural and reserves/settlements = 4

Abbreviations: CYSN: children and youth with support needs; NSS: nursing support services. Reserves/settlements: First Nation and immigrant communities.

^a^ Four participants oversaw > 1 program/service.

**FIGURE 2:**
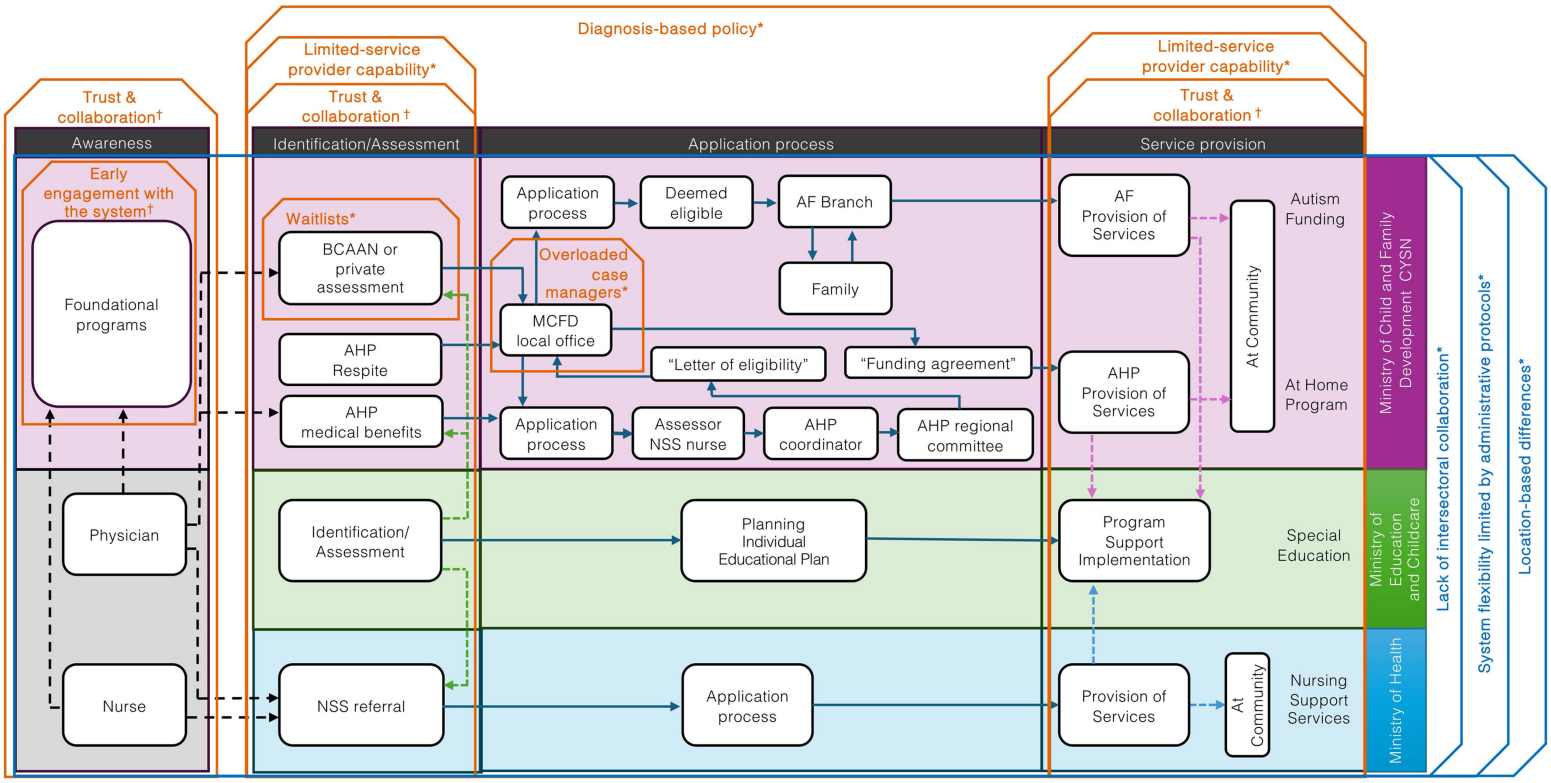
Barriers and facilitators to access disability services and support: service providers and decision‐makers' perspectives. *Note:* Vertical‐orange boxes show barriers/facilitators along navigation in the disability services and support system (Theme 1). Horizontal‐blue boxes show barriers across intersectoral navigation in the disability services and support system (Theme 2). Dashed arrows show referral trajectories; solid arrows show steps from application to access services and support. *Environmental factors as barriers to navigating the system. †Environmental factors as barriers or facilitators to navigating the system.

Barriers and facilitators were characterized by two themes: barriers and facilitators along disability services and support navigation, and barriers across ministries navigation, with nine sub‐themes: trust and collaboration, early engagement with the system, diagnosis‐based policy, limited service provider capacity and capability, waitlists, overloaded case managers, lack of intersectoral collaboration, system flexibility limited by administrative protocols, and location‐based differences (Figure 2 and Table 3).

**TABLE 3:** Barriers and facilitators. Themes, subthemes and quotes.

	Subtheme	Quotes
Theme 1. Barriers and facilitators along disability services and support navigation	1.1 Trust and collaboration	F: ‘The people who are here the most, [is] the people who have trust in our system, so that's why we are really relationship‐based.’ (P06) F: ‘As they transition out, we hope that we have supported that family to be confident in themselves and their knowledge of their child, be assertive enough to be empowered to ask for what they need.’ (P02) B: ‘[…] So, if we have [children] and families who are hesitant for historical reasons to attend [services], sometimes we get pushback about our services.’ (P06) B: ‘Families have to really figure it out. If they are lucky, they'll have a therapist or a nursing support services person that will help them make those decisions.’ (P01)
	1.2 Early engagement with the system	B: ‘[…] families can be quite desperate if we are not involved, because otherwise it's just their GP [General Practitioner] and maybe a specialist, but there's no one day to day that can assist with their child being integrated into society, it depends on what programs that they are involved with.’ (P09) B: ‘Might not know that […] once their child turns three, you can get diapers paid for.’ (P01) F/B: ‘Until a provider of some kind informs them, like a family doctor or a specialist physician, like a pediatrician of some kind, or the school district.’ (P08)
	1.3 Diagnosis‐based policy	B: ‘… we know you need a diagnosis to access these more specialized services’, but ‘it does not have to be a diagnosis […] there can be really complex needs.’ (P12) B: ‘It can be hard for families to get the diagnosis your child needs, leaving the child waiting to maybe get the services and support. Families maybe still are struggling or need supports, but then they cannot access anything.’ (P12) B: ‘To access CYSN, […] you need a diagnosis of autism, not a functional need that is a behavioural phenotype of autism, that's a huge barrier. You need to have some type of physical impairment that they would accept as somebody needing support needs.’ (P08) B: ‘Criteria is quite strict. If you can hold a spoon and get maybe a bit of yogurt in your mouth, then you can eat. And “well, that kid still actually needs support to eat”.’ (P01) B: ‘How service allocation is done is different than how funding applications are done.’ (P07)
	1.4 Limited‐service provider capacity	B: ‘I do not know exactly what they do [programs for children and youth with special needs] as a whole program, but they do offer some services.’ (P09) B: ‘[…] when we do our teacher training, for instance, we get only a few courses around neurodiversity and educating the neurodiverse learner.’ (P16) B: ‘[…] a lot of GPs just do not feel that they have the skills or the knowledge. So how do we help primary care physicians?’ (P09) B: ‘[…] In terms of the middle years, six to eighteen, […] outside of school, there are very few services, and there are even fewer inclusive afterschool services.’ (P10) B: ‘Some […] districts do not even have a learning resource teacher, let alone access to a school psychologist on a regular basis or an OT or a PT that can build the plans for students who need those plans, access to SLP services or even a board‐certified behaviour consultant.’ (P07) B: ‘School boards increase the number of children in the classrooms and often do not increase staffing support. So, it's very imbalanced.’ (P16) B: ‘[…] the schools aren't ready for the kids, so that becomes a barrier.’ (P02) B: ‘There's a staff shortage in specialized therapies, so the recruitment and retention, and of course, because of a lack of that specific professional resource, then there's no access to the families to be able to access the resources.’ (P03) B: ‘Across the province and the country probably. Everybody could use more people.’ (P07) B: ‘Staff nurses experience moral distress when they have to turn someone down, and sometimes they feel like they are the only person out there.’ (P09)

	1.5 Waitlist	B: ‘It's a resource issue at the CYSN side […]’ (P14) B: ‘[…] have families on waitlist for three years.’ (P14) B: ‘Do not have enough money to support all those waitings, so we can have 200, 300 families waiting for those resources.’ (P10) B: ‘CYSN is not adequately serving the existing populations [which] just defeats the whole idea of early intervention and front‐loading the resources here.’ (P14)
1.6 Overloaded case manager	B: ‘[…] a lot of families are heavily reliant on their CYSN social worker. If a family's child does have a diagnosis, you are eligible to work with a CYSN social worker, who […] can provide a bit more support to the family, specific to their needs.’ (P12) B: ‘Because of the worker shortage, because of staffing turnover, social workers […] just have really high caseloads.’ (P12) B: ‘If you had fewer families and you could have a relationship with each of those families, you could help families address the challenges they are having before they get huge.’ (P15) B: ‘In CYSN, the social worker is almost like a broker of services.’ (P15)
Theme 2. Barriers across ministries' navigation	2.1 Lack of intersectoral collaboration	B: ‘We've over the years had high‐level, inter‐ministerial protocols that have been developed. I doubt they often make it out of the deputy's office down to the ground. Systems are still very siloed and focused on their own sort of mandates.’ (P10) B: ‘To the children and the youth with special needs program under MCFD, that does not seem to be good integration. There are other child and youth programs for people with disabilities, but they seem to exist as islands unto themselves.’ (P09)
	2.2 System flexibility limited by administrative protocols	B: ‘[…] health practitioners […] write letters of justification for equipment. They send it to the Ministry of Family and Children. A health professional says, “this is what the child needs”. And then the ministry of family and children say, “no, we do not provide that”.’ (P01) B: ‘Education cannot influence that waitlist because that's health.’ (P03) B: ‘[…] if you do not have that organizational flexibility, it's very easy to chop off services and just say, “it's not our job”.’ (P09)
	2.3 Location‐based differences	B: ‘Services and supports in the lower mainland and in southern Vancouver Island […] are perhaps simpler and easier to access than they are if you live in a northern district, even in a larger city like Fort St. John. But if you live in a small community like Burns Lake, it's going to be even harder regardless of what your designation or diagnosis is.’ (P07) B/F: ‘Are smaller [school] districts who have an amazing support staff team and they have amazing access to services, and they have worked really cooperatively and collaboratively with places like their early childhood development centers that are MCFD supported, [with] wraparound supports that you would expect to see in a larger area.’ (P07) B: ‘Do not necessarily get the services if they are in a small rural community where there is not an ASL interpreter even.’ (P05) B: ‘[…] the program is so far away that they [service providers] have a hard time geographically to get to the family to help them, to provide supports.’ (P03) B: ‘Being flexible if you are living in isolation and you do not have the same kind of access to supports, then how do you be flexible and being able to offer different versions of extra support?’ (P03)

Abbreviations: B: barrier. F: facilitator.

The policy and program design recommendations were captured in two themes (Policy: a ‘wider door’ and Holistic System: ‘works for everybody’) with six sub‐themes (functional eligibility criteria and outcomes, life course perspective, funding, intersectoral collaboration, system navigation, training) (Table 4).

**TABLE 4:** Policy and system recommendations. Themes, subthemes and quotes.

	Subtheme	Quotes
Theme 1. Policy: a ‘wider door’ (P01)	1.1 Functional eligibility criteria and outcomes	‘Functional as opposed to diagnostic‐based eligibility criteria.’ (P08) ‘Because those [social skills] are the most important once you leave school anyway, for everyone.’ (P13) ‘To open the door wider to services for more kids. And then, once you get in the door, they need to look closer at individual circumstances and needs and create more flexible policies that allow for individualized support.’ (P01) ‘Program agency‐wide evaluations [aligned] to the framework of quality of life.’ (P14)
	1.2 Life course perspective	‘To have the extra support throughout the child's lifespan […] because the child is familiar with the one consultant and the trust and all of that is already built up.’ (P03) ‘Through developmental milestones.’ (P09) ‘For change and transitions to occur.’ (P03)
	1.3 Funding	‘Increase government funding through education, health […].’ (P16) ‘Seeing some sort of structure that was provided by the provincial government to say, “When you are in this community, we know that you need this type of increase. So, […] not only here's the funding for you to decide, we expect you to show us you have an increase of representation in your buildings or an increase of access to specialists”.’ (P06) ‘Simplifying the diagnostic criteria where there's still service efficacy there would really help and some additional resources and dollars put into that.’ (P14) ‘So that potentially small [school] districts that are hard to travel to, could have an increased funding for positions for specialists so that we can have those more accessible.’ (P06) ‘Investment into addressing wait lists.’ (P14)
Theme 2. Holistic system: ‘works for everybody’ (P01)	2.1 Intersectoral collaboration	‘Cross‐ministry collaboration for services.’ (P01) ‘[…] a formal review of gaps in care by the Ministry of Health, the Ministry of Children and Family Development, Ministry of Education, to examine where the gaps are, and to figure out ways to partner around those gaps.’ (P09) ‘Good working relationships between the ministries and everyone involved with the job [could] make the transition easier.’ (P12) ‘Lead agency model, where you can reduce the administration expenses regionally.’ (P13)

	2.2 System navigation	*Partnership* ‘[…] trying to ensure that there's perhaps better communication around the different roles within government and different roles within our co‐governance system.’ (P07) ‘[…] the communication between entities would be helpful so that if somebody's bringing in a specialist, we can create relationships throughout the entity so we can share those costs or share those resources.’ (P06) ‘Staff who actually are willing to do community development instead of just sitting behind a desk.’ (P13) ‘[…] school district with almost 200 different languages spoken […], we thought, “how can we support those families that have multiple barriers to accessing a regular family doctor, let alone a pediatrician?” We created that team to do those diagnoses.’ (P05) ‘[…] the production of short video clips that talk about […] some of the key messages that we want to communicate in their language. And we need to do more on that front. We're just at the tip of the iceberg in terms of figuring out how to communicate with the changing demographics here.’ (P14) *Long‐term case manager* ‘The importance of holding the person's story, but if you do not have any case management function, it's hard because you need that one person to help hold that story as they go from child and youth to adults.’ (P09) ‘Need help navigating the system, or if they need help administering the [support] plans, then we need people who are very knowledgeable in those areas trained to help families do that.’ (P01) ‘Make the paperwork that families have to manage simpler […], particularly around [At Home Program] and around accessing autism funding.’ (P15) ‘Creating a more flexible schedule or a more flexible access route to the social worker or to the services. […] might make it a little easier for families to access.’ (P15) ‘Do not know how to reach [families], but the ministry can reach them because they have that information. They could look at who the kids are on the [CYSN] program and see who is not accessing services.’ (P01) *Data collection* ‘If a kid [has] at‐home program funding, but that family has never submitted anything for physio, OT or speech, they [CYSN] should be finding out why. And they should be figuring out who those families are. Those are families that are missing out completely on support for therapy. There [are] ways to find out what's missing and to make the system work for everybody.’ (P01) ‘[…] when we get students who transfer from other schools, sometimes we do not get the records of services they have received or how many schools they have attended. […] So, we have our student records fractured, so having a clearer history of education would be helpful.’ (P06)
2.3 Training	‘More education for both the people running MCFD, but also our community partners around what we [do]. Because if there was more understanding, […] within the schools or within the doctors or pediatricians, that would've helped grease the kids for more families to connect.’ (P15) ‘That everybody knows what's available and can support people to get there.’ (P15) ‘Sometimes the frontline staff are too narrow about their criteria, so you have to ask for a supervisor. I think that maybe we need to train that staff differently so that that's not the case.’ (P01)

### 3.1 Barriers and Facilitators

Participants suggested that, while some facilitators might promote access to services and support, children and their families had to overcome numerous barriers to increase their access to disability services and navigate the education, health and social service system. Findings are described and organized within the disability system navigation in two themes: (1) from awareness of the existence of services and support to service provision, and (2) across cross‐sectoral provision of services and support (Figure 2 and Table 3).

#### 3.1.1 Theme 1. Barriers and Facilitators Along Disability Services and Support Navigation

##### 3.1.1.1 Trust and Collaboration

The quality of the connections and relationships between the education and disability system's staff and the families is relevant, particularly in the awareness, identification/assessment and service provision phases of the disability system navigation (Figure 2).

According to participants (Table 3), trust and collaboration are important facilitators of access to services. Participants believe that families experience trust in the system when a family‐centred approach is used, and providers invest time and resources to support children and their families. Moreover, families supported in accessing services and support experience a reciprocal, positive interaction with providers. This facilitates families' access to knowledge and information in the awareness, identification/assessment and service provision phases, empowering them to advocate for themselves and their needs. Conversely, participants described that a family's lack of trust in the system and low collaboration between service provider organizations and families are barriers for all families navigating siloed services and support, particularly for those who have faced negative interactions with child protection services.

##### 3.1.1.2 Early Engagement Before School Age With the System

Early engagement with the system before school age could be a barrier or a facilitator for awareness of existing services and support (Figure 2). Participants agreed (Table 3) that families and caretakers of children engaged in early development programs are more likely to have better early access to publicly available information about applying for funding and firsthand advice and support from frontline professionals working in the disability system. On the contrary, not being engaged with early development programs before school age limits access to timely information regarding the services and support available in the system.

##### 3.1.1.3 Diagnosis‐Based Policy

An education and disability policy based on diagnostic criteria for receiving funding to access services and support narrows the chances of a family accessing funding for services and support in a timely manner. Participants described disability policies as a barrier in the identification and assessment phases, and by extension, in the application process and service provision phases (Figure 2).

As described by participants (Table 3), requiring a specific diagnosis to access funding rather than using need‐based criteria has two consequences. Children with impairments or functional and behavioural needs for support that do not qualify as a specific, eligible diagnosis do not receive services and support until they obtain a confirmed diagnosis. Families will also be ineligible to access funding if they do not meet the specific criteria for a funding source. Moreover, the diagnosis‐based policy dictates that service provision in schools varies according to the funding source. Overall, children sharing similar needs but different diagnoses would have unequal access to funding sources and different amounts of funding depending on the program they are eligible for.

##### 3.1.1.4 Limited‐Service Provider Capacity and Capability

Barriers relevant to the identification/assessment and service provision phases include service providers' knowledge, the availability of staff and programs, and moral distress (Figure 2). Some participants emphasized (Table 3) that workers from different levels in organizations that provide disability services and support have limited knowledge (capacity) about the system's programs and their purpose. Additionally, a lack of financial resources limits the system's capability to provide ongoing staff training, which is particularly relevant in the identification phase. The system's financial constraints diminish its capability to provide disability programs, resulting in a shortage of specialized staff for school‐based and community‐based support. This becomes critical in schools, which are perceived as not prepared to support children with special needs. Finally, some participants reported experiences of moral distress among frontline workers when confronted with the challenges families face while navigating an under‐resourced system, lacking the power to counterbalance the difficulties that families and their children encounter.

##### 3.1.1.5 Waitlists

Waitlists are considered a barrier in the identification/assessment phase, particularly for diagnosis screening in the Autism Funding program (Figure 2). As described in Table 3, participants indicated that the long waitlists relate to a ‘resource issue’ centralized at the ministry level. Children on waitlists struggle due to behavioural needs, and service provider organizations do not have the financial capacity to support them while waiting to be eligible. Consequently, CYSN is perceived as not serving the population.

##### 3.1.1.6 Overloaded Case Managers

Many participants emphasized that an overloaded case manager role becomes a barrier in the application process (Figure 2). Some participants explained (Table 3) that an overloaded case manager or social worker lacks the time and support to build connections with families. The consequence is that case managers are perceived as distant and uninvolved workers, often associated with a resource or funding management profile.

#### 3.1.2 Theme 2. Barriers Across Ministries' Navigation

##### 3.1.2.1 Lack of Intersectoral Collaboration

Silos and a lack of intersectoral collaboration are barriers that challenge families navigating the ministries (Figure 2). As some participants described (Table 3), inter‐ministerial protocols have been developed but poorly communicated, creating gaps across the system that impact how programs in health and education relate to each other.

##### 3.1.2.2 System Flexibility Limited by Administrative Protocols

Participants agreed that administrative protocols challenge families and service providers alike by limiting the overall system's flexibility to provide services and support across ministries (Figure 2). In most cases (Table 3), the policies regulating disability funding programs translate into administrative protocols tied to a specific diagnosis for the provision of services and support. Service providers often lack policy mechanisms that enable them to provide services or support tailored to the needs of families and children, regardless of whether they have a formal diagnosis.

##### 3.1.2.3 Location‐Based Differences

Participants agreed that challenges associated with the barriers and facilitators vary depending on the location where the family lives and where services are provided, related to the geographical and population diversity in the province (Figure 2). In addition, as indicated in Table 3, barriers and facilitators will also vary depending on jurisdictional differences between health regions and school districts in the province, regardless of the geographical extension. This implies that a factor acting as a barrier for a family in a health region or school district might not be so in another jurisdiction, or it could even be a facilitator. Moreover, these differences also apply between rural and urban settings, within and across health regions and school districts. Families and service providers are impacted by a shortage of specialized professionals in rural communities, as well as the challenges that system protocols and long distances pose to service providers in delivering services and support.

### 3.2 Policy and System Recommendations

To address the barriers and leverage facilitators, the participants recommend policy changes to enable a ‘wider door’ (P01) for eligibility and access to disability services and support, along with changes aimed at building a holistic system that ‘works for everybody’ (P01). These recommendations are organized into two themes (Table 4). Changes in the navigation process from awareness to providing services and support are addressed by Themes 1 and 2 (functional eligibility criteria and outcomes, life course perspective, funding, system navigation and training), and changes across ministries are addressed by Theme 2 (intersectoral collaboration).

#### 3.2.1 Theme 1. Policy: A ‘Wider Door’ (P01)

##### 3.2.1.1 Functional Eligibility Criteria and Outcomes

Participants recommended (Table 4) wider eligibility criteria to access funding, services and support by shifting to functional eligibility criteria, strengthening a more flexible, social‐based policy and allowing for individualized support. A Quality of Life framework could also guide the achievement of functional and social outcomes, as well as program evaluations.

##### 3.2.1.2 Life Course Perspective

Participants agreed that a policy with a life course perspective acknowledges the changing needs of families and children, guiding the provision of support throughout the child's lifespan (Table 4). A life course perspective would also guide the coordination of services and support during the navigation process.

##### 3.2.1.3 Funding

Participants suggested (Table 4) that governmental financial resources should be modified and tailored to address the gaps identified across the disability system and to meet local needs for services and support. Changes should aim to modify the allocation of financial resources across health regions and school districts to improve the system's capacity to deliver services and support and to address waitlists.

#### 3.2.2 Theme 2. Holistic System: ‘Works for Everybody’ (P01)

##### 3.2.2.1 Intersectoral Collaboration

A crucial recommendation is to improve cross‐ministry collaboration at all levels within the system (Table 4). This would enable transitioning from a siloed to an interconnected structure and organization to provide disability services and support. Collaboration and strong working relationships among all parties involved in every step of service provision across ministries, along with a lead agency model, could enhance system efficiency.

##### 3.2.2.2 System Navigation

Participants recommended changes to improve system navigation by promoting partnerships and facilitating collaborations across the system, using a language and culturally informed approach. Changes should also secure a long‐term case manager role and systematic data collection (Table 4).

###### 3.2.2.2.1 Partnerships

Most participants recommended building partnerships across organizations that provide services and support to improve system navigation (Table 4). Organizations sharing costs or resources could address the limited‐service provider capability gaps. Partnerships should also be built between people working in the system and families seeking services and support in a community development approach. Participants agreed that partnerships in the system could support navigation by developing services and support adapted to families' and children's cultural needs and challenges.

###### 3.2.2.2.2 Long‐Term Case Manager

Participants emphasized that a critical improvement in the system is securing a long‐term case management position (Table 4). In addition to centralizing information, a long‐term case manager role should establish a collaboration‐based relationship with the family. To support the role of the case manager, it will be necessary to simplify the administrative process for eligibility and accessing services and support, as well as include a person‐centred approach. Triage protocols can also support the case manager's decision‐making process. Information collected about families and children's characteristics and needs should be used to prioritize access to disability funding, services and support based on their needs.

###### 3.2.2.2.3 Data Collection

Some participants agreed that systematic data collection on children and families is necessary to inform decision‐making at multiple levels within the disability system (Table 4). Data collection can support navigation by identifying families not accessing services and support and facilitating transitions between programs when families move within the province.

##### 3.2.2.3 Training

Participants recommended training at all levels across the disability system to promote a holistic system, including topics such as system structure and organization (Table 4). Updates on health and functional conditions, diagnostics, assessments and interventions, as well as equity, diversity and inclusion, can help strengthen the knowledge and skills of frontline staff.

## 4 Discussion

This study aimed to describe the perspectives of service providers and decision‐makers about disparities in access to education, health and social disability services and support in bc for children and youth with NDD and their families. Participants described barriers and facilitators and provided recommendations for policy and program design to decrease disparities in eligibility and improve access.

Findings show that policy and disability services and support implementation determine a ‘narrow’ door for children with NDD and their families to be eligible and access the disability system. Additionally, system navigation encompasses multiple barriers and facilitators along and across the fragmented system of services and support.

Building trust and promoting collaboration between staff and families, along with early family engagement in the system, can serve as facilitators in most steps of system navigation. Additional system characteristics include barriers along and across the disability support and service navigation system. These barriers include diagnosis‐based policies, limited service provider capacity and capability, waitlists for diagnosis, overloaded case managers, a lack of intersectoral collaboration, administrative protocols that restrict system flexibility and discrepancies in the allocation of financial resources and service provision based on location.

Our findings align with current literature on barriers and facilitators to access NDD services and support in education, health and social sectors from high‐, middle‐ and low‐income countries, as reported by children with NDD, their families and service providers (Edwards et al. 2022; Finlay, Wittevrongel, et al. 2023; Clemente et al. 2022; Teleman et al. 2021; Sapiets et al. 2021; Gardiner et al. 2022; Xie et al. 2024; Batz and Yadav 2024; Magnusson et al. 2022; Matthews et al. 2021; Moran and Sheppard 2023; Zuckerman et al. 2017; Bear et al. 2020; Harrison et al. 2023; Khanlou et al. 2015).

Previous studies have reported trust and collaboration between staff and families as barriers or facilitators (Clemente et al. 2022; Teleman et al. 2021; Sapiets et al. 2021; Gardiner et al. 2022; Xie et al. 2024; Batz and Yadav 2024; Matthews et al. 2021; Moran and Sheppard 2023; Khanlou et al. 2015). Service providers' beliefs and attitudes toward the family and children can promote or hinder mutual trust and collaboration, leading to higher or lower levels of self‐confidence in the family to actively engage with the system (Teleman et al. 2021) at different points in the navigation process (Sapiets et al. 2021). Simultaneously, timely and early engagement with the disability system may increase the opportunities to build relationships and access information and support to navigate the system (Moran and Sheppard 2023).

Our study emphasized two critical barriers to accessing services and support previously reported in the literature: waitlists for diagnosis during the identification/assessment stage (Edwards et al. 2022; Sapiets et al. 2021; Gardiner et al. 2022; Moran and Sheppard 2023; Bear et al. 2020) and overloaded case managers during the application process (Matthews et al. 2021; Bear et al. 2020). Disparities emerging from waitlists are related to a diagnosis‐based funding policy that generates differences in the opportunity to receive a diagnosis and access services and support among neurodevelopmental conditions (Sapiets et al. 2021; Gardiner et al. 2022; Matthews et al. 2021; Moran and Sheppard 2023; Zuckerman et al. 2017; Bear et al. 2020). Once the child is diagnosed, accessing services may be delayed in a burdensome application process with varying requirements and paperwork (Finlay, Wittevrongel, et al. 2023) and centralized in an overloaded case management role. The role of a case manager has been described as a crucial element for navigational support in the disability system (Miller et al. 2023), managing the application process and, most importantly, providing various types of support to families, as well as facilitating and coordinating access to resources in the disability system (Miller et al. 2023).

The system's capability to deliver disability services and support in the identification/assessment and service provision stages, or its lack thereof, has been reported as a systemic barrier at different levels of care across health, educational and social sectors (Clemente et al. 2022; Sapiets et al. 2021; Gardiner et al. 2022; Xie et al. 2024; Magnusson et al. 2022; Matthews et al. 2021; Moran and Sheppard 2023; Bear et al. 2020; Khanlou et al. 2015). In our study, limited service provider capacity and capability are described related to various aspects of service implementation (knowledge capability, staffing, availability of programs, financial resources), primarily due to a funding structure that restricts the system's financial capacity.

In conjunction with the aforementioned factors along the navigation process, our study identified three additional barriers across the disability system. Lack of intersectoral collaboration is a systemic barrier posing additional challenges for navigation, deepening the gaps in education within a disability system with disconnected and disparate services (Matthews et al. 2021). This barrier is pervasive across all stages of the navigation process, exacerbating the disparities described previously. Studies have reported that a lack of intersectoral collaboration between organizations is a critical barrier (Magnusson et al. 2022) to accessing health and rehabilitation services for persons with disabilities. By extension, it limits the chances of building a relationship based on trust between service providers and families (Khanlou et al. 2015), as well as between service providers in different organizations (Clemente et al. 2022), which negatively impacts navigation and challenges families in their advocacy capability to surpass the gaps in the system (Matthews et al. 2021). One of the many roles of a case manager or care coordinator is to bridge services across disparate systems (Gardiner et al. 2022), a function that would be limited in an overloaded case manager.

System flexibility limited by administrative protocols was reported as another important barrier. This barrier relates to the policies regulating disability funding programs, which hinder the provision of equitable services tailored to the needs of families and children, and the system's capability to adapt. Previous studies have reported that system flexibility is a relevant factor in enabling access across services and support at all stages of the navigation process by delivering services at accessible locations, times and modalities for families (Clemente et al. 2022; Sapiets et al. 2021; Gardiner et al. 2022; Xie et al. 2024; Magnusson et al. 2022; Khanlou et al. 2015).

All barriers and facilitators impact equitable access and system navigation differently at different phases and vary according to the demographic, geographical and, more importantly, the jurisdictional context. Few studies have reported location‐based differences as barriers to accessing services and support (Finlay, Wittevrongel, et al. 2023; Sapiets et al. 2021), mainly geographical accessibility and its interaction with transportation and distance to services (Xie et al. 2024). Our findings align with a previous pan‐Canadian study reporting that the number and type of programs available vary by location (Finlay, Wittevrongel, et al. 2023), attributed to jurisdictional differences across ministries by participants in our study.

To our knowledge, this study is the first to report contextual differences in how system environmental factors challenge the navigation process within the education, health and social disability system of services and support. This is an important finding, as it contributes to disparities that families and their children face when accessing the disability system at all stages of the navigation process across the province, particularly for families and their children and youth with NDD aged 0–18. Furthermore, our study presents a conjoint and cross‐sectoral analysis of service providers' and decision‐makers' perspectives relative to the disparities in access to disability services and support in bc. Participants identified systemic barriers and facilitators, framing a comprehensive system perspective of the challenges that families encounter when interacting with the disability system.

Participants recommended policy and program implementation changes to improve eligibility and access to services and support. Policy improvement should aim to attain a ‘wider door’ to eligibility and access. This could be achieved by shifting to functional eligibility criteria and functional outcomes to assess program implementation at a population level. This should be complemented by a life course perspective that is transferred from policy to service delivery and coordination, enabling the system to adapt to the changing needs of the child and the family over time. A ‘wider door’ would require modifying the amount and allocation of governmental financial resources channelled through different funding programs, tailored to the gaps identified in the disability system.

Participants also recommended solutions to enable a ‘holistic’ disability system that ‘works for everybody’. Improving intersectoral collaboration across ministries and all stages of the navigation process is critical. This should be complemented by improvements in system navigation through fostering partnerships between organizations, utilizing a language and culturally informed approach, securing a long‐term case manager role and supporting the system with up‐to‐date data collection. Finally, training across the disability system aimed at building service provider capacity to promote a holistic system.

Few previous studies have reported solutions to barriers identified from the service providers' perspectives. Those recommendations relate to a caseworker model and continuing education for service providers (Khanlou et al. 2015), promoting partnerships across the disability system (Matthews et al. 2021), providing general recommendations to improve access within the early intervention system of services and support (Sapiets et al. 2021), implementing an integrative model of navigation (Gardiner et al. 2022), designing programs with a family‐focused approach (Batz and Yadav 2024), utilizing data analysis and a multiperspective engagement of people delivering and receiving services to design recommendations (Moran and Sheppard 2023) and peer‐to‐peer family navigation services along with improvements to the infrastructure supporting the application process (Bear et al. 2020).

This study presents recommendations for policy and implementation improvements aimed at the overall structure of the disability system of services and support for children and youth with NDDs, from an integrated perspective of service providers and decision‐makers working in the disability system.

The results from this study should be considered in light of its limitations. Themes identified in the analysis reflect the perspectives of a sub‐group of service providers and decision‐makers contacted during recruitment and who agreed to be interviewed, mainly working under CYSN and NSS jurisdictions with a lesser representation of MEdC services and support, which may not be representative of all the people working in the disability system across the province. This study used the province of bc, Canada, to answer the research question and objectives, and our results might not apply to other contexts. The perspectives of families and children with NDD were not part of the scope of this study but have been captured previously (Finlay, Wittevrongel, et al. 2023). Nonetheless, we acknowledge the value and role their perspective plays in policy and implementation improvements in the disability system of services and support.

## 5 Conclusions

Findings from this study provide a systemic and intersectoral overview of the interrelated challenges that arise while navigating the system of disability services and support and apply to many jurisdictions. Complex and fragmented disability services and support systems pose multiple barriers to eligibility and access to health, educational and social support. The barriers and facilitators intertwine within the system before, during and after the disability system establishes interaction with families and their children who seek the services and support they need. It creates a complex setting to deliver services, hampering an equitable provision of disability services and support.

This study identifies recommendations to address the implementation gap between the provincial provision of services and Canadian commitments to the United Nations Convention on the Rights of Persons with Disabilities to facilitate full participation in society for individuals with disabilities. Findings can be applied to other jurisdictions. However, solutions should be constructed on evidence acknowledging the existence of a large contextual, jurisdictional, geographical and demographical variability if equitable access to services and support were to be implemented. Barriers should be addressed, and facilitators could be leveraged at a provincial level through the recommendations described in this study to promote equitable access to disability services and support for children with NDD and their families.

## Author Contributions

**Patricia Basualto:** methodology, data curation, investigation, formal analysis, writing – original draft, review and editing, visualization. **Angela M. Senevirathna:** investigation, formal analysis. **Ashish** **Seth:** investigation. **Gina Dimitropoulos:** and **Jennifer D. Zwicker**funding acquisition, conceptualization, methodology, supervision, validation, formal analysis, writing – review and editing. All authors reviewed and approved the submitted version of the manuscript.

## Funding

The authors gratefully acknowledge the contributions from Kids Brain Health Network funded through The Strategic Science Fund, Canadian Institute for Health Research (PJT‐173325) and the Sinneave Family Foundation. Jennifer D. Zwicker is supported by a Tier II Canada Research Chair in Disability Policy for Children and Youth. Patricia Basualto is supported by ANID/Doctorado Becas Chile 72220050. The funders had no role in study design, data collection and analysis, publication decisions or manuscript preparation.

## Ethics Statement

The research project ‘ACCESS Assessing the Continuum of Care and Eligibility for Services and Supports for Children with Neurodevelopmental Disabilities and their Families’ was reviewed and approved through the Conjoint Faculties Research Ethics Board from the University of Calgary (REB 20‐1872 and REB 21‐1597). All participants provided individual consent to participate. Guidelines for reporting qualitative research of the study can be found in Supporting Information S1.

## Consent

The authors have nothing to report.

## Conflicts of Interest

The authors declare no conflicts of interest.

## Supporting information


**Data S1** Supporting Information.

## Data Availability

The data that support the findings of this study are available on request from the corresponding author. The data are not publicly available due to privacy or ethical restrictions.
